# Supplementary figures and images for: Community knowledge, attitudes and practices related to Taenia solium taeniosis and cysticercosis in Zambia
Source: PLoS Negl Trop Dis. 2023 Aug 10;17(8):e0011375. doi: 10.1371/journal.pntd.0011375 (PMC10443877; doi:10.1371/journal.pntd.0011375)

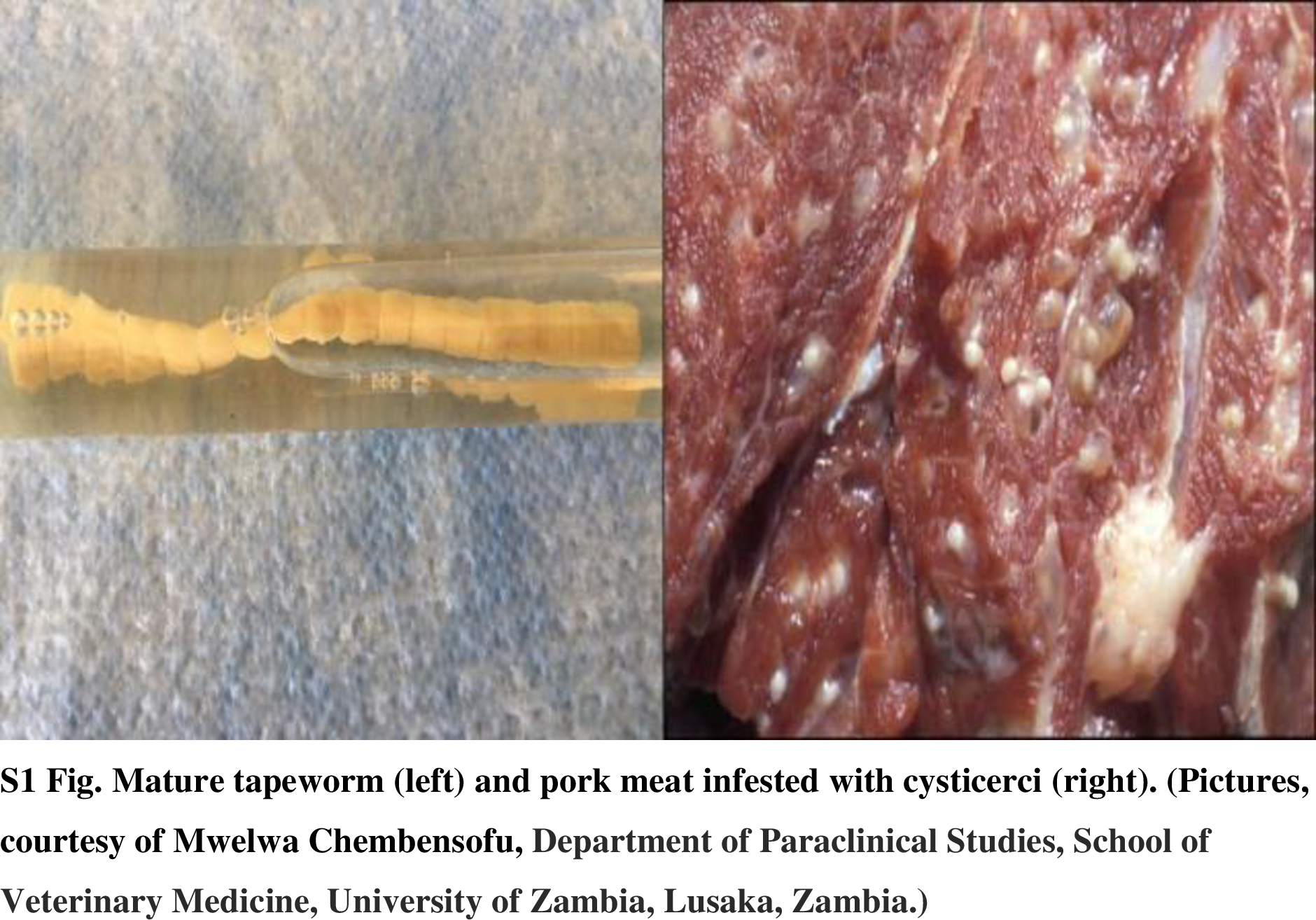

Supplement: S1 Fig — (TIF) [file pntd.0011375.s002.tif]
