# Supplementary material for: Community knowledge, attitudes and practices related to Taenia solium taeniosis and cysticercosis in Zambia
Source: PLoS Negl Trop Dis. 2023 Aug 10;17(8):e0011375. doi: 10.1371/journal.pntd.0011375 (PMC10443877; doi:10.1371/journal.pntd.0011375)
Supplement: S1 File — (DOC) [file pntd.0011375.s003.doc]

**S1 FILE: STUDY HOUSEHOLD QUESTIONNAIRE**

**(To be administered to each participating household)**

| **HH CODE** |  |
| --- | --- |

| **Questionnaire** | |
| --- | --- |
| NOTE: Interviewer please fill in the space provided and circle all responses from participants | |
| General Information | |
| 1. Date of interview *(dd/mm/yyyy)* |  |
| 1. District |  |
| 1. Village |  |
| 1. Household number |  |
| 1. Name of household head |  |
| 1. Name of interviewer |  |
| Personal Information of The Respondent | |
| 1. Position of interviewee in the household | 1. Head of household 2. Spouse 3. Dependant 4. Other (Please specify)_______________ |
| 1. Age |  |
| 1. Sex | - - 1. Male     2. Female     3. Other (please specify) _____________ |
| 1. Religion | 1. Catholic 2. Protestant 3. Adventist 4. Muslim 5. Other (Please specify) ________________ |
| 1. What is the highest level of education you completed? | 1. None 2. Primary school 3. Secondary school 4. Tertiary |
| 1. What is your main occupation? | 1. Farmer 2. Trader 3. Student 4. Formal employment 5. None 6. Other (Please specify) ______________ |
| 1. What is your main source of income? | 1. Farming 2. Business 3. Formal employment 4. Other (please specify) ______________ |
| Information on Drinking Water and Sanitation | |
| 1. Is access to water a problem for your household? | 1. Yes 2. No |
| 1. From where do you usually get your drinking water? | 1. River 2. Well 3. Bore-hole 4. Tap 5. Other (Please specify): __________ |
| 1. How do you treat your water before drinking? | 1. Boiling 2. Add Chlorine 3. Not treated 4. Other (please specify) |
| 1. Do you have a latrine at home? | 1. Yes 2. No (If No, go to Q23) |
| 1. Is the latrine used? | 1. Yes 2. No (If No, go to Q20) 3. Sometimes |
| 1. If yes check for evidence of use (Physical inspection) | 1. Yes 2. No |
| 1. What materials is the latrine made from? (Physical inspection ) | 1. Burnt brick wall 2. Mud and logs wall 3. Thatched grass wall 4. Iron sheets roof 5. Grass roof 6. No roof |
| 1. Does the latrine have a door? (Physical inspection) | 1. Yes 2. No |
| 1. Is there a hand washing facility near the latrine? (Physical inspection) | 1. Yes 2. No |
| 1. Do you always wash your hands after using the toilet? | 1. Yes, always 2. Sometimes 3. No, never (If No, go to Q25) |
| 1. What do you use to wash hands after using the toilet? | 1. Water only 2. Water with soap 3. Water with ashes 4. Do not wash |
| 1. Do you share the latrine with neighbours? | 1. Yes 2. No |
| 1. Do you have a raised dish rack? (physical inspection) | 1. Yes 2. No |
| **IV. Awareness of Human Tapeworm Infections** | |
| 1. Have you ever heard of tapeworm infections in humans? | 1. Yes 2. No (If No, go to section V) 3. Not sure (If not sure, go to section V) |
| 1. How did you learn about it? | a. Hospital  b. Family/friends  c. School  d. Other (please specify)______________ |
| 1. How does one know they have tapeworm infection? | 1. Abdominal pain 2. Vomiting 3. Diarrhoea 4. Seen in faeces 5. Don’t know |
| 1. How does one acquire tapeworm infection? | 1. Eating contaminated food 2. Drinking contaminated water 3. Eating infected pork meat 4. Not washing hands after using the toilet 5. Don’t know 6. Others (specify)___________________ |
| 1. How can one prevent him/herself from acquiring tapeworm infection? | 1. Properly cooking their pork meat 2. Washing hands after using toilet 3. Washing hands before eating 4. Discarding contaminated pork meat 5. Other (please specify)­­­­­­­­­­­___________ |
| 1. What should people with tapeworm infection do? | 1. Go to the hospital 2. Go to a traditional healer 3. Do nothing 4. Don’t know 5. Other (specify)_____________________ |
| 1. Do you think you are at risk of infection with tapeworm? | 1. Yes 2. No (If No, go to Q35) 3. Don’t know |
| 1. If ‘Yes’ why do you think you are at risk of acquiring tapeworm infection? |  |
| 1. If ‘No’ why do you think you are not at risk of acquiring tapeworm infection? |  |
| **V. Awareness of Human Cysticercosis Infections** | |
| 1. Have you ever head of human cysticercosis (HCC)? | 1. Yes 2. No (If No, go to section VI) |
| 1. How did you learn about it? | 1. Hospital 2. Family/friends 3. School 4. Other (please specify)______________ |
| 1. How does one acquire human cysticercosis? | 1. Eating undercooked infected pork 2. Eating unwashed fruits and vegetables 3. Eating food contaminated with tapeworm eggs 4. Not washing hands after defecating and before eating 5. Don’t know 6. Other (please specify) ______________ |
| 1. Where can lesions for human cysticercosis be located? | 1. Brain 2. Eyes 3. Skin 4. Muscle 5. Don’t know 6. Other (please specifiy) _______________ |
| 1. Can human cysticercosis cause serious health problems? | 1. Yes 2. No 3. Don’t know |
| 1. What are the symptoms of human cysticercosis | 1. Chronic headaches 2. Epilepsy/ seizures 3. Subcutaneous nodules 4. Abdominal pain 5. Paralysis 6. Don’t know 7. Other (please specify) |
| 1. How can one suspect they have human cysticercosis in the brain (NCC)? | 1. Chronic headaches 2. Epilepsy/ seizures 3. Blurred vision 4. Paralysis 5. Don’t know 6. Other (please specify) _____________ |
| 1. How can one prevent him/herself from getting human cysticercosis? | 1. Properly cooking their pork meat 2. Washing hands after using toilet 3. Washing hands before eating 4. Discarding contaminated pork meat 5. Don’t know 6. Other (please specify) ­­­­­­­­­­­___________ |
| 1. What should people with human cysticercosis do? | 1. Go to the hospital 2. Go to a traditional healer 3. Do nothing 4. Don’t know 5. Other (specify)_____________________ |
| 1. Do you think you are at risk of cysticerci infection? | 1. Yes 2. No (If No, go to Q46) 3. Don’t know |
| 1. If ‘Yes’ why do you feel you are at risk of cysticerci infection? |  |
| 1. If ‘No’ why do you feel you are not at risk of cysticerci infection? |  |
| VI. Information on Pork Consumption and Management | |
| 1. Do you or anyone in your household keep pigs? | 1. Yes 2. No (If No, go to Q50) |
| 1. How many pigs do you have? |  |
| 1. How do you manage your pigs? | 1. Free ranging 2. Permanently housed 3. Housed at night 4. Tethered 5. Other (specify)_______________ |
| 1. Do you or any member of your household eat pork meat? | 1. Yes 2. No (If No, go to Q54)) |
| 1. How often do you eat pork meat? | 1. At least once a month 2. More than once a month 3. Less than once a month but at least once a year 4. Less than once a year |
| 1. How is the pork that you eat prepared? | 1. Boiled 2. Fried 3. Barbeque 4. Other (specify) _______________ |
| 1. Where do you usually get the pork that you eat | 1. From own stock 2. From local market 3. From commercial butchery 4. Other (specify)_______________ |
| 1. Have you ever slaughtered a pig at home? | a. Yes  b. No (If No, go to section VII) |
| 1. Was the pork inspected the last time you slaughtered a pig at home? | 1. Yes 2. No |
| 1. If not inspected what was the reason for not inspecting the pork? | 1. No inspectors available 2. No money for inspection 3. No reason for inspection 4. Other (please specify) ____________ |
| **VII. Awareness of Porcine Cysticercosis** |  |
| 1. Have you ever observed a pig being slaughtered in your neighbourhood? | 1. Yes 2. No |
| 1. Have you ever seen cysts (“*masese*”) in pork? | 1. Yes 2. No (If No, go to Q60 |
| 1. What was done to the pork with cysts *(‘masese’)* that you saw? | 1. Was sold 2. Was eaten 3. Was discarded 4. Don’t know 5. Other (please specify) |
| 1. Do you know what cysts *masese*) are? | 1. Yes 2. No ( If No, skip to section VIII) |
| 1. If ‘Yes’ what do you think cysts *(masese*) are? |  |
| 1. What are the locations for cysts (*masese*)in pigs? | 1. Muscle 2. Under the tongue 3. Brain 4. Heart 5. Stomach 6. Neck muscles 7. Other (please specify) ________________ |
| 1. Do you know how pigs acquire the cysts (*masese)*? | 1. Yes 2. No (If No, go to Q65) |
| 1. If ‘Yes’ how do pigs acquire cysts (*masese*)? | 1. Feeding on contaminated food/water 2. Eating contaminated human faeces 3. Other (Please specify)_________________ |
| 1. When you see cysts (*masese)* in pork meat do you eat the meat? | 1. Yes 2. No |
| 1. When you see cysts (*masese)* in pork meat do you sell the meat? | 1. Yes 2. No |
| 1. When you see cysts (*masese)* in pork meat do you discard the meat? | 1. Yes 2. No |
| 1. Is there a way to prevent pigs from getting cysticercosis (masese)? | 1. Yes 2. No 3. Don’t know |
| 1. If ‘Yes’ how can pigs be prevented from getting cysticercosis (masese)? | 1. Raising pigs in a pigpen 2. Humans always using toilets 3. Other (please specify) ____________ |
| 1. **Epilepsy Perception** |  |
| 1. Have you heard about epilepsy? | 1. Yes 2. No (If No, End Interview) |
| 1. Have you heard of anyone with epilepsy in your village? | 1. Yes 2. No (If No, go to Q75) |
| 1. Do you have anyone with epilepsy in your household? | 1. Yes 2. No (If No, go to Q75) |
| 1. If “Yes” did they received treatment? | 1. Yes 2. No (If No, go to Q75) |
| 1. If ‘Yes’ Where did they go for treatment? | 1. Hospital 2. Traditional healer 3. Spiritual prayers 4. Don’t know 5. Other (please specify) __________ |
| 1. Do you know what causes epilepsy? | 1. Yes 2. No (If No, go to Q79) |
| 1. If ‘Yes’ what causes epilepsy? |  |
| 1. Can epilepsy be caused by human cysticercosis ? | 1. Yes 2. No 3. Don’t know |
| 1. If ‘yes’ how can epilepsy be caused by human cysticercosis? |  |
| 1. Can epilepsy be transmitted from one person to another? | 1. Yes 2. No (If No, go to Q81) 3. Don’t know (If Don’t know, go to Q81) |
| 1. If ‘Yes’ how can epilepsy be transmitted from one person to another? |  |
| 1. Can epilepsy be transmitted from pigs to humans? | 1. Yes 2. No 3. Don’t Know |
| 1. If ‘Yes’ how can epilepsy be transmitted from pigs to humans? |  |
| 1. Do you consider epilepsy to be a serious disease? | 1. Yes 2. No (If No, go to Q83) |
| 1. If ‘Yes’, why would you consider epilepsy to be a serious disease? |  |
| 1. What should people with epilepsy do? | 1. Go to the hospital 2. Go to a traditional healer 3. Go for prayers 4. Do nothing 5. Don’t know 6. Other (please specify) __________ |
| 1. What is your perception of people with epilepsy? |  |
| **We have come to the end of the questionnaire. I wish to thank you most sincerely for sparing some time to answer these questions; your participation is greatly appreciated** | |
