# Supplementary material for: Community knowledge, attitudes and practices related to Taenia solium taeniosis and cysticercosis in Zambia
Source: PLoS Negl Trop Dis. 2023 Aug 10;17(8):e0011375. doi: 10.1371/journal.pntd.0011375 (PMC10443877; doi:10.1371/journal.pntd.0011375)
Supplement: S2 File — (PDF) [file pntd.0011375.s004.pdf]

S2 File. Community knowledge, attitudes and practices related to *Taenia solium* taeniosis and cysticercosis in Zambia.

Table A. Knowledge about Pork tapeworm infection (human *T. solium* taeniosis)

Question	Total (n)	Response	Number	Percent
Have you ever heard of tapeworm infections in humans?	588	Yes	351	60
		No	194	33
		Not sure	43	7
How did you learn about it?	351	Hospital	107	30
		Family/friends	60	17
		School	7	2
		Other	177	50
If "other" specify	177	Through community sensitization	163	92
		Have seen them before	11	6
		Radio	3	2
How does one acquire tapeworm infection?	351	Eating contaminated food	98	28
		Drinking contaminated water	19	5
		Eating infected pork meat	57	16
		Not washing hands after using the toilet	36	10
		Don’t know	100	28
		Other	41	12
How can one prevent him/herself from acquiring tapeworm infection?	351	Properly cooking their pork meat	135	38
		Washing hands after using toilet	89	25
		Washing hands before eating	22	6
		Discarding contaminated pork meat	3	1
		Other	102	29
What should people with tapeworm infection do?	351	Go to the hospital	348	99.1
		Don’t know	3	0.9

Table B. Knowledge about Human Cysticercosis

Question	Total (n)	Response	Number	Percent
Have you ever head of human cysticercosis (HCC)?	588	Yes	334	57
		No	254	43
How did you learn about it?	334	Hospital	78	23
		Family/friends	29	9
		Other	227	68
If "other" specify	227	Community sensitization	226	99.6
		Town during business	1	0.4
How does one acquire human cysticercosis?	334	Eating undercooked infected pork	238	71
		Eating unwashed fruits and vegetables	5	1
		Eating food contaminated with tapeworm eggs	4	1
		Not washing hands after defecating and before eating	9	3
		Don't know	70	21
		Other	8	2
Where can lesions for human cysticercosis be located?		Brain	107	32
		Eyes	1	0
		Skin	42	13
		Muscle	65	19
		Don`t Know	77	23
		Other	42	13
What are the symptoms of human cysticercosis		Chronic headaches	61	18
		Epilepsy/ seizures	30	9
		Subcutaneous nodules	3	1
		Abdominal pain	25	8
		Don't know	197	59
		Other	18	5
How can one suspect they have human cysticercosis in the brain (NCC)?		Chronic headaches	66	21
		Epilepsy/ seizures	60	18
		Blurred vision	4	1
		Paralysis	1	0
		Don't know	185	55
		Other	18	5
How can one prevent him/herself from getting human cysticercosis?		Properly cooking their pork meat	144	43
		Washing hands after using toilet	11	3
		Washing hands before eating	6	2
		Discarding contaminated pork meat	17	5
		Don't know	108	32
		Other	48	14
What should people with human cysticercosis do?		Go to the hospital	314	94
		Don't know	20	6

Table C. Knowledge about porcine cysticercosis

Question	Total (n)	Response	Number	Percent
Do you know what cysts masese) are?	588	Yes	411	70
		No	177	30
What do you think cysts (masese) are?	411	Infection in pigs	226	55
		White watery things in pork meat	154	37
		They're Small Blisters in pork meat	3	1
		Bacteria	11	3
		They are maggots in pork meat	17	4
What are the locations for cysts (masese) in pigs?		Muscle	172	42
		Under the tongue	198	48
		Stomach	9	2
		Neck muscles	6	1
		Other	26	6
Do you know how pigs acquire the cysts (masese)?		Yes	172	42
		No	239	58
How do pigs acquire cysts (masese)?	172	Feeding on contaminated food/water	45	26
		Eating contaminated human faeces	64	37
		Other	63	37
If "other" specify	63	Feeding on gaga left over from kachasu beer	50	79
		Feeding on gaga (Maize bran)	9	14
		From the mud on the ground	1	2
		Feeding of infected feed	1	2
		Some pigs are born with cysts	1	2
		Pigs eating salt	1	2
Is there a way to prevent pigs from getting cysticercosis (masese)?	411	Yes	209	51
		No	6	1
		Don't know	196	48
How can pigs be prevented from getting cysticercosis (masese)?	209	Raising pigs in a pigpens	160	77
		Humans always using toilets	19	9
		Other	30	14
If "other" specify	30	Stop feeding the pigs gaga from kachasu beer	12	40
		Give them clean feed	4	13
		Give medicines to protect the pigs	14	47

Table D. Perceptions / Knowledge about Epilepsy

Question	Total (n)	Response	Number	Percent
Have you heard about epilepsy?	588	Yes	566	96
		No	22	4
Do you know what causes epilepsy?	566	Yes	46	8
		No	520	92
what causes epilepsy?	46	Witchcraft	8	17
		Eating infected pork meat	14	30
		Infections like malaria	14	30
		It runs in families	10	22
Can epilepsy be caused by human cysticercosis ?	566	Yes	67	12
		No	65	11
		Don't know	434	77
Can epilepsy be transmitted from one person to another?		Yes	41	7
		No	374	66
		Don't know	151	27
How can epilepsy be transmitted from one person to another?	41	Don't Know exactly	2	5
		When one inhales flatus from a convulsing person	25	61
		If you have sex with a person with epilepsy	2	5
		Through families e.g. parents to children	7	17
		If you touch a person during convulsions	5	12
Can epilepsy be transmitted from pigs to humans?	566	Yes	89	16
		No	126	22
		Don't know	351	62
How can epilepsy be transmitted from pigs to humans?	89	If you eat infected pork meat	89	100
Do you consider epilepsy to be a serious disease?	566	Yes	510	90
		No	56	10
What should people with epilepsy do?		Go to the hospital	560	99
		Go to a traditional healer	1	0
		Other	5	1
What is your perception of people with epilepsy?		They are normal like everyone	93	16
		They are not normal	87	15
		They need care and support	97	17
		They have a mental problem	99	17
		They have a serious disease	79	14
		I feel sorry for them	68	12
		They have high risk of injuries and dying	43	8

Table E. Attitudes towards human tapeworm infection (*T. solium* taeniosis) and human cysticercosis and porcine cysticercosis

Question	Total (n)	Response	Number	Percent
Do you think you are at risk of infection with tapeworm?	351	Yes	136	39
		No	32	5
		Don't know	183	31
Why do you think you are not at risk of acquiring tapeworm infection?	32	Does not have any signs of the infection	6	19
		I keep my house and surrounding clean	6	19
		I don't eat soil	11	34
		It's a disease for children	2	6
		Always wash hands before eating	4	13
		I boil pork	1	3
		Was recently treated for worms	2	6
Do you think you are at risk of cysticerci infection?	334	Yes	116	35
		No	38	11
		Don't know	180	54
Why do you feel you are not at risk of cysticerci infection?	38	Have no signs of infection	4	11
		Always wash hands after toilet	6	16
		I don't eat infected pork	24	63
		Just feel like not at risk	4	11
When you see cysts (masese) in pork meat do you eat the meat?	411	Yes	26	6
		No	385	94
When you see cysts (masese) in pork meat do you sell the meat?		Yes	53	13
		No	358	87
When you see cysts (masese) in pork meat do you discard the meat?		Yes	374	91
		No	37	9

Table F. Information on Pigs

Question	Total (n)	Response	Number	Percent
Do you or anyone in your household keep pigs?	588	Yes	98	17
		No	490	83
How do you manage your pigs?	98	Free ranging	54	55
		Permanently housed	27	28
		Housed at night	17	17
Do you or any member of your household eat pork meat?	588	Yes	420	71
		No	168	29
How is the pork that you eat prepared?	420	Boiled	400	95
		Fried	10	2
		Barbeque	10	2
Have you ever slaughtered a pig at home?	588	Yes	108	18
		No	480	82
Was the pork inspected the last time you slaughtered a pig at home?	108	Yes	11	10
		No	97	90
If not inspected what was the reason for not inspecting the pork?	97	No inspectors available	21	22
		No money for inspection	8	8
		No reason for inspection	65	67
		Other	3	3
If "other" specify	3	Not aware of need for inspection	3	100
Have you ever observed a pig being slaughtered in your neighbourhood?	588	Yes	324	55
		No	264	45
Have you ever seen cysts (“masese”) in pork?		Yes	517	88
		No	71	12
What was done to the pork with cysts (‘masese’) that you saw?	517	Was sold	191	37
		Was eaten	101	20
		Was discarded	202	39
		Don’t know	18	3
		Other	5	1

Table G. Sanitation and Hygiene

Question	Total (n)	Response	Number	Percent
From where do you usually get your drinking water?	588	Well	36	6.1
		Bore-hole	550	93.5
		Tap	2	0.3
How do you treat your water before drinking?		Boiling	7	1.2
		Add Chlorine	39	6.6
		Not treated	542	92.2
Do you have a latrine at home?		Yes	422	71.8
		No	166	28.2
Is the latrine used?	422	Yes	421	99.8
		No	1	0.2
Is there a hand washing facility near the latrine? (Physical inspection)		Yes	49	11.6
		No	373	88.4
Do you always wash your hands after using the toilet?	588	Yes, always	419	71.3
		Sometimes	160	27.2
		No, never	9	1.5
What do you use to wash hands after using the toilet?	579	Water only	274	47.3
		Water with soap	255	44.0
		Water with ashes	50	8.6

Table H. Significant differences in responses between males and females

Question	Options	Sex				Total		χ^2 test
		Male	%	Female	%	n	%	p-value
Do you have a latrine at home?	Yes	198	76%	224	68%	422	72%	0.027
	No	61	24%	105	32%	166	28%	
Do you think you are at risk of cysticerci infection?	Yes	54	39%	62	32%	116	35%	0.033
	No	21	15%	17	9%	38	11%	
	Don't know	64	46%	116	60%	180	54%	
Do you or any member of your household eat pork meat?	Yes	197	76%	223	68%	420	71%	0.028
	No	62	24%	106	32%	168	29%	
Have you ever slaughtered a pig at home?	Yes	73	28%	35	11%	108	18%	< 0.001
	No	186	72%	294	89%	480	82%	
Have you ever seen cysts ("masese") in pork?	Yes	236	91%	281	85%	517	88%	0.041
	No	23	9%	48	15%	71	12%	
Do you know what cysts masese) are?	Yes	193	75%	218	66%	411	70%	0.037
	No	66	26%	111	34%	177	30%	
Do you know how pigs acquire the cysts (masese)?	Yes	92	48%	80	37%	172	42%	0.028
	No	101	52%	137	63%	238	58%	
Can epilepsy be transmitted from one person to another?	Yes	22	9%	19	6%	41	7%	< 0.001
	No	184	73%	190	60%	374	66%	
	Don't know	45	18%	106	34%	151	27%	
Can epilepsy be transmitted from pigs to humans?	Yes	46	18%	43	14%	89	16%	0.024
	No	65	26%	61	19%	126	22%	
	Don't know	140	56%	211	67%	351	62%	

Significance level set at $p = 0.05$
